# Brain pericytes among cells constituting the blood-brain barrier are highly sensitive to tumor necrosis factor-α, releasing matrix metalloproteinase-9 and migrating *in vitro*

**DOI:** 10.1186/1742-2094-8-106

**Published:** 2011-08-26

**Authors:** Fuyuko Takata, Shinya Dohgu, Junichi Matsumoto, Hiroyuki Takahashi, Takashi Machida, Tomoya Wakigawa, Eriko Harada, Haruki Miyaji, Mitsuhisa Koga, Tsuyoshi Nishioku, Atsushi Yamauchi, Yasufumi Kataoka

**Affiliations:** 1 Department of Pharmaceutical Care and Health Sciences, Faculty of Pharmaceutical Sciences, Fukuoka University, Fukuoka, Japan; 2 BBB Laboratory, PharmaCo-Cell Co., Ltd., Nagasaki, Japan; 3 Academic, Industrial and Governmental Institute for Aging and Brain Sciences, Fukuoka University, Fukuoka, Japan

## Abstract

**Background:**

Increased matrix metalloproteinase (MMP)-9 in the plasma and brain is associated with blood-brain barrier (BBB) disruption through proteolytic activity in neuroinflammatory diseases. MMP-9 is present in the brain microvasculature and its vicinity, where brain microvascular endothelial cells (BMECs), pericytes and astrocytes constitute the BBB. Little is known about the cellular source and role of MMP-9 at the BBB. Here, we examined the ability of pericytes to release MMP-9 and migrate in response to inflammatory mediators in comparison with BMECs and astrocytes, using primary cultures isolated from rat brains.

**Methods:**

The culture supernatants were collected from primary cultures of rat brain endothelial cells, pericytes, or astrocytes. MMP-9 activities and levels in the supernatants were measured by gelatin zymography and western blot, respectively. The involvement of signaling molecules including mitogen-activated protein kinases (MAPKs) and phosphoinositide-3-kinase (PI3K)/Akt in the mediation of tumor necrosis factor (TNF)-α-induced MMP-9 release was examined using specific inhibitors. The functional activity of MMP-9 was evaluated by a cell migration assay.

**Results:**

Zymographic and western blot analyses demonstrated that TNF-α stimulated pericytes to release MMP-9, and this release was much higher than from BMECs or astrocytes. Other inflammatory mediators [interleukin (IL)-1β, interferon-γ, IL-6 and lipopolysaccharide] failed to induce MMP-9 release from pericytes. TNF-α-induced MMP-9 release from pericytes was found to be mediated by MAPKs and PI3K. Scratch wound healing assay showed that in contrast to BMECs and astrocytes the extent of pericyte migration was significantly increased by TNF-α. This pericyte migration was inhibited by anti-MMP-9 antibody.

**Conclusion:**

These findings suggest that pericytes are most sensitive to TNF-α in terms of MMP-9 release, and are the major source of MMP-9 at the BBB. This pericyte-derived MMP-9 initiated cellular migration of pericytes, which might be involved in pericyte loss in the damaged BBB.

## Background

Brain pericytes are located adjacent to capillaries and share a common basement membrane with brain microvascular endothelial cells (BMECs). This allows pericytes to communicate directly with BMECs through gap junctions and peg-and-socket contacts to stabilize microvessels and regulate cerebral blood flow by their contractile and relaxant properties [1-3]. Along with BMECs and astrocytes, pericytes constitute the blood-brain barrier (BBB), and communicate with BMECs through release of soluble factors, leading to the up-regulation of BBB functions [4-8]. Recently, it has been reported that BBB breakdown and hypoperfusion occurs in viable pericyte-deficient mice [9,10], suggesting that brain pericytes play a crucial role in BBB integrity and cerebral microcirculation under healthy conditions. Furthermore, the genetic animal models of progressive pericyte loss with age have shown that BBB integrity is determined by the extent of pericyte coverage of cerebral microvessels [9]. Thus, BBB dysfunction is attributed to brain pericyte loss in the microvasculature.

Pericyte loss or reduced pericyte coverage has been observed in several pathological animal models. We demonstrated that detachment of brain pericytes from the basal lamina occurs in disruption of the BBB, caused by lipopolysaccharide (LPS)-induced sepsis in mice [11]. In cerebral ischemia, which induces BBB disruption [12], the detachment and migration of brain pericytes were observed [13]. These findings suggest that these pericyte behaviors are involved in BBB disruption. It has been reported that brain pericytes extend toward the parenchyma, and the basal lamina becomes thin in the early stage of brain hypoxia [14] and traumatic injury [15]. These morphological alterations were interpreted as the initial step of pericyte migration [16]. In this step, pericytes appear to exhibit high proteolytic activities.

Matrix metalloproteinases (MMPs), a family of zinc-dependent endopeptidases, are expressed in pericytes to degrade the components of the extracellular matrix under physiological conditions. Elevated levels of MMP-9 in brain with cerebral ischemia [17,18] are closely associated with BBB disruption [19,20]. In BMECs, astrocytes, microglia and neurons, MMP-9 production is stimulated by proinflammatory cytokines including tumor necrosis factor (TNF)-α. TNF-α, a known mediator of neuroinflammation, is produced by brain insults such as stroke. BBB permeability and MMP-9 expression in the brain microvessels were increased in obese mice with stroke [21]. These findings raise the possibility that brain microvessels rather than brain parenchyma are the major source of MMP-9.

To test whether MMP-9 production and subsequent migration of pericytes contribute to BBB disruption associated with neuroinflammation, we examined the ability of pericytes to release MMP-9 and migrate in response to TNF-α, and compared it with that of BMECs and astrocytes.

## Methods

### Materials

Dulbecco's modified Eagle's medium (DMEM) and DMEM/Ham's nutrient mixture F-12 medium (DMEM/F12) were purchased from Wako (Osaka, Japan) and Sigma (St. Louis, MO, USA), respectively. Fetal bovine serum (FBS) and plasma-derived serum (PDS) were purchased from Biowest (Nuaillé, France) and Animal Technologies Inc. (Tyler, TX, USA), respectively. TNF-α was from R&D systems Inc. (Minneapolis, MN, USA). U0126, SP600125, SB203580 and LY294002 were from Tocris (Ellisville, MO, USA).

### Cell culture

All procedures involving experimental animals were conducted in accordance with the law (No. 105) and notification (No.6) of the Japanese Government, and were approved by the Laboratory Animal Care and Use Committee of Fukuoka University.

Primary cultures of rat brain pericytes and rat brain microvascular endothelial cells (RBECs) were prepared from three-week-old Wistar rats, as previously described [1,4,22,23]. The meninges were carefully removed from forebrains, and the gray matter was minced in ice-cold DMEM and digested with collagenase type 2 (1 mg/mL, Worthington, Lakewood, NJ, USA) for 1.5 h at 37°C. The pellet was separated by centrifugation in 20% bovine serum albumin (BSA)-DMEM (1000 × g, 20 min). The microvessels obtained in the pellet were further digested with collagenase/dispase (1 mg/mL, Roche, Mannheim, Germany) for 1 h at 37°C. Microvessel clusters containing pericytes and endothelial cells were separated on a 33% continuous Percoll (GE Healthcare, Buckinghamshire, UK) gradient, collected and washed twice with DMEM before plating on non-coated dishes and collagen type IV-fibronectin (both 0.1 mg/mL) coated dishes. Brain pericyte cultures were maintained in DMEM supplemented with 20% FBS and 50 μg/mL gentamicin (Biowest). After seven days in culture, pericytes at 80-90% confluency were used for experiments. RBEC cultures were maintained in RBEC medium Ι [DMEM/F12 supplemented with 10% PDS, basic fibroblast growth factor (1.5 ng/mL, Roche), heparin (100 μg/mL, Sigma), insulin (5 μg/mL), transferrin (5 μg/mL), sodium selenite (5 ng/mL; insulin-transferrin-sodium selenite media supplement, Sigma) and gentamicin (50 μg/mL)] containing puromycin (4 μg/mL, Sigma) at 37°C in a humidified atmosphere of 5% CO_2_/95% air, for two days. To remove the puromycin, cells were washed three times with fresh RBEC medium Ι and incubated with this medium on the third day. On the fifth day, RBECs typically reached 80-90% confluency.

Primary astrocyte cultures were prepared from the cerebral cortex of one- to three-day-old Wistar rats according to the method of McCarthy and de Vellis (1980) [24] with a slight modification. Briefly, after removing the meninges and blood vessels, the forebrains were minced and gently dissociated by repeated pipetting in DMEM containing 10% FBS, 100 units/mL penicillin (Nacalai Tesque, Kyoto, Japan) and 100 μg/mL streptomycin (Nacalai Tesque), and filtered through a 70-μm cell strainer. Cells were collected by centrifugation (800 × g, 6 min), resuspended in 10% FBS DMEM and cultured in 75-cm^2 ^flasks (BD Biosciences, Franklin Lakes, NJ, USA) in a humidified atmosphere of 5% CO_2_/95% air at 37°C. Cells were fed every 2-3 days by changing medium. After 10-14 days in culture, floating cells and weakly attached cells of the mixed primary cultured cell layer were removed by vigorous shaking of the flask. Then, astrocytes at the bottom of the culture flask were trypsinized and seeded into new culture flasks. The primary cultured astrocytes were maintained in 10% FBS/DMEM. They were grown in a humidified atmosphere of 5% CO_2_/95% air at 37°C. Cells at the second or third passage were used for experiments.

### Western blot analysis

Brain pericytes, astrocytes and RBECs were incubated with or without different concentrations of TNF-α at 37°C for the indicated time. When protein kinase inhibitors were used, they were added 15 min prior to the application of TNF-α. To compare the expression of TNF-α receptor 1 (TNFR1) and TNF-α receptor 2 (TNFR2) among brain pericytes, astrocytes and RBECs, these cells were used without TNF-α treatment. The culture supernatants were collected and concentrated ~60-fold using Amicon^® ^Ultra centrifugal filter devices (cut-off MW: 30 kDa, Millipore Corporation, Billerica, MA, USA). Cells were scraped and lysed in phosphoprotein lysis buffer (10 mM Tris-HCl, pH 6.8, 100 mM NaCl, 1 mM EDTA, 10% glycerol, 1% Triton X-100, 0.1% SDS, 0.5% sodium deoxycholate, 2 mM Na_3_VO_4_, 50 mM NaF, 20 mM sodium pyrophosphate decahydrate and 50 μg/mL phenylmethylsulfonyl fluoride) containing 1% phosphatase inhibitor cocktail 1 (Sigma), 1% phosphatase inhibitor cocktail 2 (Sigma) and 1% protease inhibitor cocktail (Sigma). The total protein concentration in cell lysates was determined using a BCA Protein assay kit (Pierce, Rockford, IL, USA). Equivalent amounts of protein from each sample were electrophoretically separated on 5-20% SDS-polyacrylamide gels (Bio Craft Co., Ltd., Tokyo, Japan), and then transferred to polyvinylidene difluoride membranes (Bio-Rad Laboratories, Hercules, CA, USA). Membranes were blocked with Blocking One (Nacalai Tesque) or Blocking One-P (Nacalai Tesque) for phosphorylated proteins. Phosphorylation of p42/p44 mitogen-activated protein kinase (MAPK), p38 MAPK, c-Jun N-terminal kinase (JNK) and Akt were detected with primary antibodies against phospho-p42/p44 MAPK (1:2000; Cell Signaling Technology, Danvers, MA, USA), phospho-p38 MAPK, phospho-JNK and phospho-Akt (1:1000; Cell Signaling Technology). MMP-9 and MMP-2 in culture supernatant were detected using antibodies against MMP-9 (1:500; Millipore Corporate) and MMP-2 (1:500; R&D Systems Inc.). TNFR1 and TNFR2 in cell lysates were detected with an anti-MMP-9 antibody (1:200; Santa Cruz Biotechnology Inc., Delaware, CA, USA) and anti-MMP-2 antibody (1:1000; Cell Signaling Technology). After washing, membranes were incubated with an appropriate horseradish peroxidase-conjugated secondary antibody. To reprobe total p42/p44 MAPK, p38 MAPK, JNK and Akt, membranes were incubated in stripping buffer (0.2 M glycine, 0.1% SDS and 1% Tween 20, pH 2.2) for 15 min twice. Total p42/p44 MAPK, p38 MAPK, JNK and Akt were detected using primary antibodies against p42/p44 MAPK, p38 MAPK, JNK and Akt (1:1000; Cell Signaling Technology). The immunoreactive bands were visualized using an ECL Advance Western Blotting Detection Kit (GE Healthcare). The band images were digitally captured with a FluorChem SP imaging system (Alpha Innotech, San Leandro, CA, USA) and band intensities were quantified using AlphaEaseFC software (Alpha Innotech). The relative intensity of phosphorylation of individual proteins was expressed as the ratio of phosphorylated protein and the corresponding total protein.

### Gelatin zymography

Brain pericyte-conditioned media were concentrated by Amicon^® ^Ultra centrifugal filter devices, and then subjected to zymography according to the manufacturer's recommendations (Life laboratory company, Yamagata, Japan). Zymographic results were expressed as MMP proteolytic activity and were measured with a FluorChem SP imaging system and band intensities were quantified using AlphaEaseFC software.

### Migration assay

Rat brain pericytes, RBECs and astrocytes were seeded on collagen IV-coated center-well organ culture dishes (BD Biosciences) and cultured to confluence in 20% FBS/DMEM, RBEC medium I and 10% FBS/DMEM, respectively. Cells were scratched manually with a sterile 0.1-10 μL pipette tip, and the detached cells were removed by washing three times with serum-free DMEM or serum-free RBEC medium I. To test whether MMP-9 participates in TNF-α-induced migration of pericytes, the cells were exposed to control mouse IgG (10 μg/mL; Sigma) with 10% FBS/DMEM (vehicle) and mouse monoclonal anti-MMP-9 antibody (1:20; clone 2C3, Abcam, Cambridge, MA, USA) or control mouse IgG (10 μg/mL) with TNF-α (50 ng/mL). Astrocytes and RBECs were exposed to 10% FBS/DMEM and RBEC medium I with or without TNF-α (50 ng/mL), respectively. Then, cells were incubated for 72 h. Phase contrast images of seven to eight fixed positions in the wound area were taken at 0 and 72 h after scratching using a microscope with a built-in digital camera (Biozero, BZ-8000; KEYENCE, Osaka, Japan). In the images, the edge of the initial wound area was marked by lines using BZ-Analyzer software (KEYENCE) just before scratching. The edge of the initial wound area was overlaid with the image taken at 72 h after scratching. The number of cells migrating into the initial wound area was counted at 72 h after scratching. The data were obtained from three separate assays.

### Statistical analysis

Results are shown as means ± S.E.M. The statistical significance of differences between groups was assessed by one-way analysis of variance (ANOVA) for factorial comparisons and by Dunnett's or Tukey-Kramer's test for multiple comparisons. Differences were considered significant when *P *values were less than 0.05, using GraphPad Prism 5.0 (GraphPad, San Diego, CA, USA).

## Results

### TNF-α induces MMP-9 release from brain pericytes

**Figure 1:**
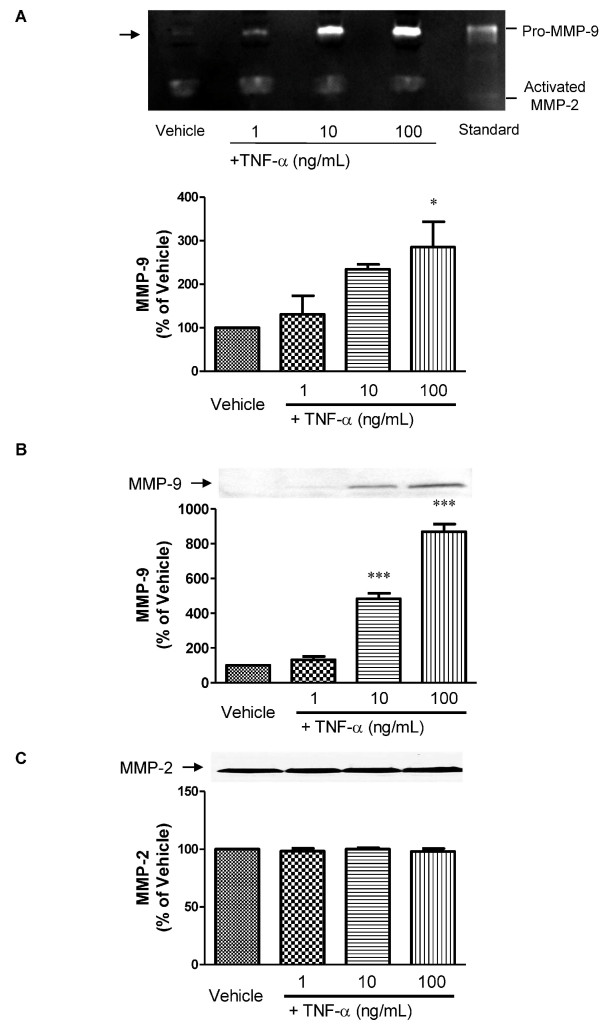
**Release of MMP-9 and MMP-2 from brain pericytes after TNF-α treatment (1, 10 and 100 ng/mL) for 24 h**. A, Representative zymograms showing MMP-9 and MMP-2 activities in the culture media of pericytes (top panel). Densitometric analysis of MMP-9 (bottom panel) and MMP-2 (data shown in the text) activities in the culture media of pericytes. B and C, Representative western blots (top panels) and densitometric analysis (bottom panels) of protein levels of MMP-9 and MMP-2, respectively, in the culture media of pericytes. Band intensities were quantified by scanning densitometry and the data are expressed as a percentage of control values (vehicle-treated pericytes). Each bar indicates mean ± S.E.M. for three experiments. * *P *< 0.05, *** *P *< 0.001, significantly different from vehicle-treated group.

Gelatin zymographic analysis revealed a band at the position approximately under the standard pro-MMP-9 band, indicating that the supernatant of the pericytes had MMP-9 activity (Figure 1A, arrowed band). A 24-h exposure to TNF-α (1, 10 and 100 ng/mL) increased MMP-9 activities in the supernatant of primary cultures of pericytes in a concentration-dependent manner (31, 135 and 185% of increase, respectively; Figure 1A). Western blot analysis using an anti-MMP-9 antibody showed that in response to TNF-α (10 and 100 ng/mL for 24 h) MMP-9 release from pericytes increased in a concentration dependent manner by 383 and 769% of vehicle, respectively (Figure 1B). These increases in the MMP-9 protein levels were consistent with the zymographic activities (Figure 1A). When TNF-α was incubated at 95°C for 5 min, this denatured TNF-α (100 ng/mL for 24 h) failed to induce MMP-9 release from pericytes (data not shown). TNF-α did not induce significant changes in MMP-2 activities (1, 10 and 100 ng/mL: 171.8 ± 66.81, 160.2 ± 70.21 and 167.7 ± 66.46% of vehicle, respectively; n = 3-4) and MMP-2 levels (Figure 1A, top panel and 1C, respectively). A 24-h exposure to TNF-α (1, 10, and 100 ng/mL) showed no effect on cell viability as determined by mitochondrial dehydrogenase activity (WST-8) assay (99.6 ± 2.93, 109.8 ± 1.67 and 107.3 ± 1.70% of vehicle, respectively; n = 4). To determine whether other inflammatory mediators induce MMP-9 release from pericytes, we treated cells with interleukin (IL)-1β, interferon (IFN)-γ, IL-6 and LPS (1, 10, and 100 ng/mL of each substance) for 24 h. None of these inflammatory mediators induced MMP-9 release from pericytes (data not shown).

### Pericytes are the major source of MMP-9 released from cells constituting the BBB in response to TNF-α

**Figure 2:**
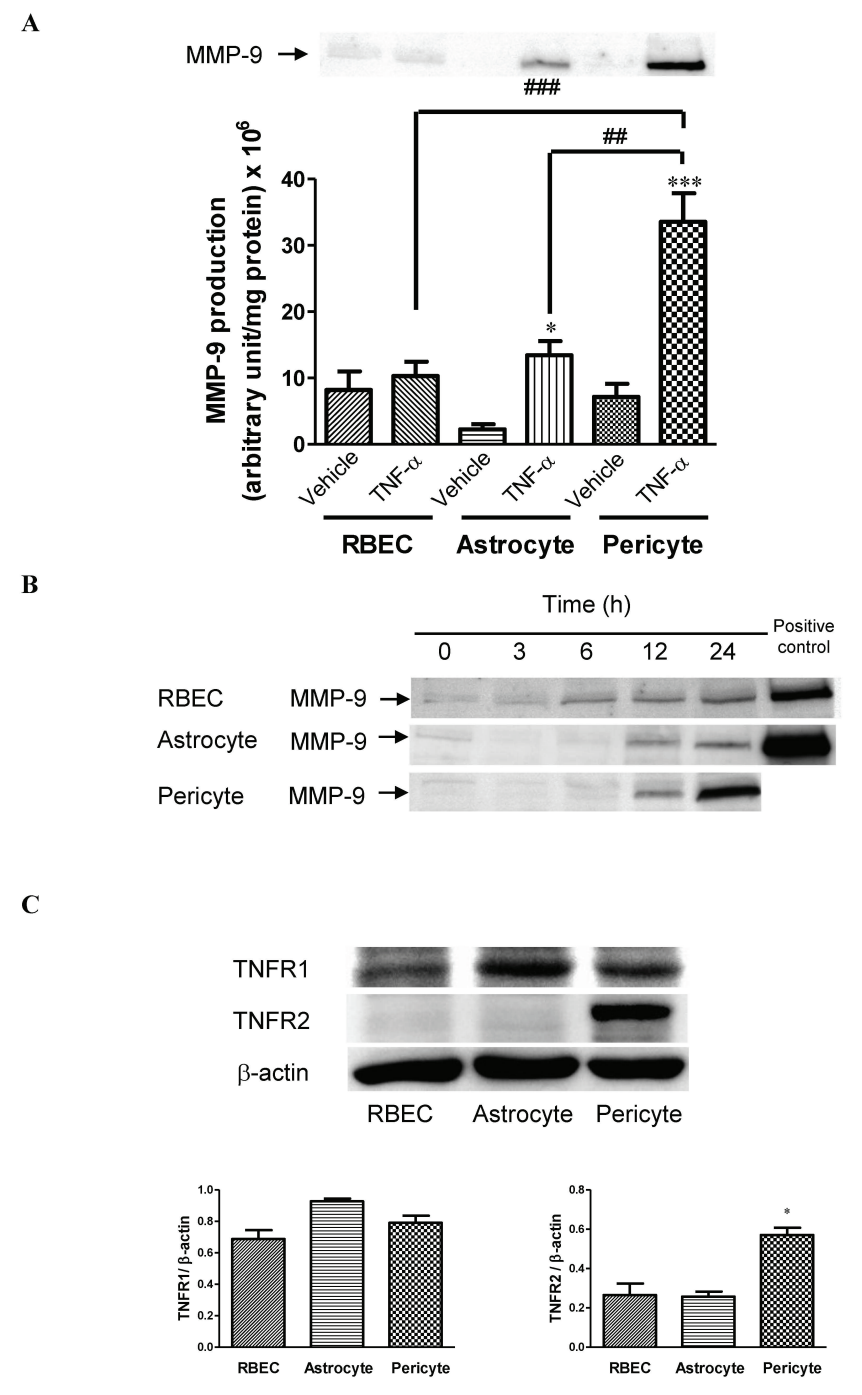
**TNF-α-induced MMP-9 release levels and the expression of TNFR1 and TNFR2 in the BBB cells RBECs, astrocytes and pericytes**. A, Representative western blots (top panel) and densitometric analysis (bottom panel) of MMP-9 levels in the culture media. Cells were treated with TNF-α (100 ng/mL) for 24 h. Band intensities were quantified by scanning densitometry and the data are expressed as arbitrary units/mg of protein. Each bar indicates mean ± S.E.M. for three experiments. * *P *< 0.05 and *** *P *< 0.001, significantly different from vehicle-treated astrocytes and pericytes, respectively. ^##^*P *< 0.01, significantly different from TNF-α-treated astrocytes. ^###^*P *< 0.001, significantly different from TNF-α-treated RBECs. B, Representative images showing the time-course of TNF-α-induced MMP-9 release from RBECs, astrocytes and pericytes. Cells were treated with TNF-α (100 ng/mL) for 3 - 24 h. MMP-9 levels in the culture media were analyzed by western blot. Levels in the culture media of TNF-α-treated pericytes were used as positive controls for levels in the media of TNF-α-treated RBECs and astrocytes. C, Representative western blots (top panels) and densitometric analysis (bottom panel) of TNFR1 and TNFR2 expression in cell lysates of non-treated RBECs, astrocytes and pericytes. Protein levels of TNFR1 and TNFR2 were normalized to β-actin and the data are expressed as a ratio relative to β-actin. Each bar indicates mean ± S.E.M. for three experiments. * *P *< 0.05, significantly different from RBECs and astrocytes.

We determined the TNF-α-induced MMP-9 release from three cellular components of the BBB (BMECs, astrocytes and pericytes) after treatment with 100 ng/mL TNF-α for 24 h. TNF-α significantly increased the release of MMP-9 from pericytes and astrocytes into the supernatant (Figure 2A). Pericytes showed marked MMP-9 release (33.6 ± 4.30 × 10^6 ^arbitrary unit/mg protein per 24 h), whereas astrocytes and RBECs produced lower levels of MMP-9 (13.4 ± 2.12 × 10^6 ^and 10.3 ± 2.16 × 10^6 ^arbitrary unit/mg protein per 24 h, respectively; Figure 2A). This TNF-α-induced MMP-9 release from pericytes was 3.3- and 2.5-fold higher than from RBECs and astrocytes, respectively. As shown in Figure 2B, TNF-α-induced release of MMP-9 from the three cell types increased with time. This increased response appeared within 12 h in each culture. As TNF-α can bind to two structurally distinct membrane receptors on target cells, TNFR1 (also known as p55 and TNFRSF1A) and TNFR2 (also known as p75 and TNFRSF1B), we examined their expression levels in RBECs, astrocytes and pericytes (Figure 2C). There were no significant differences in the expression levels of TNFR1 among RBECs, astrocytes and pericytes (Figure 2C). The expression level of TNFR2 in pericytes was about 2.2-fold higher than in RBECs and astrocytes.

### TNF-α induces MMP-9 release from pericytes via the p42/p44 MAPK, JNK, and p38 MAPK pathways

**Figure 3:**
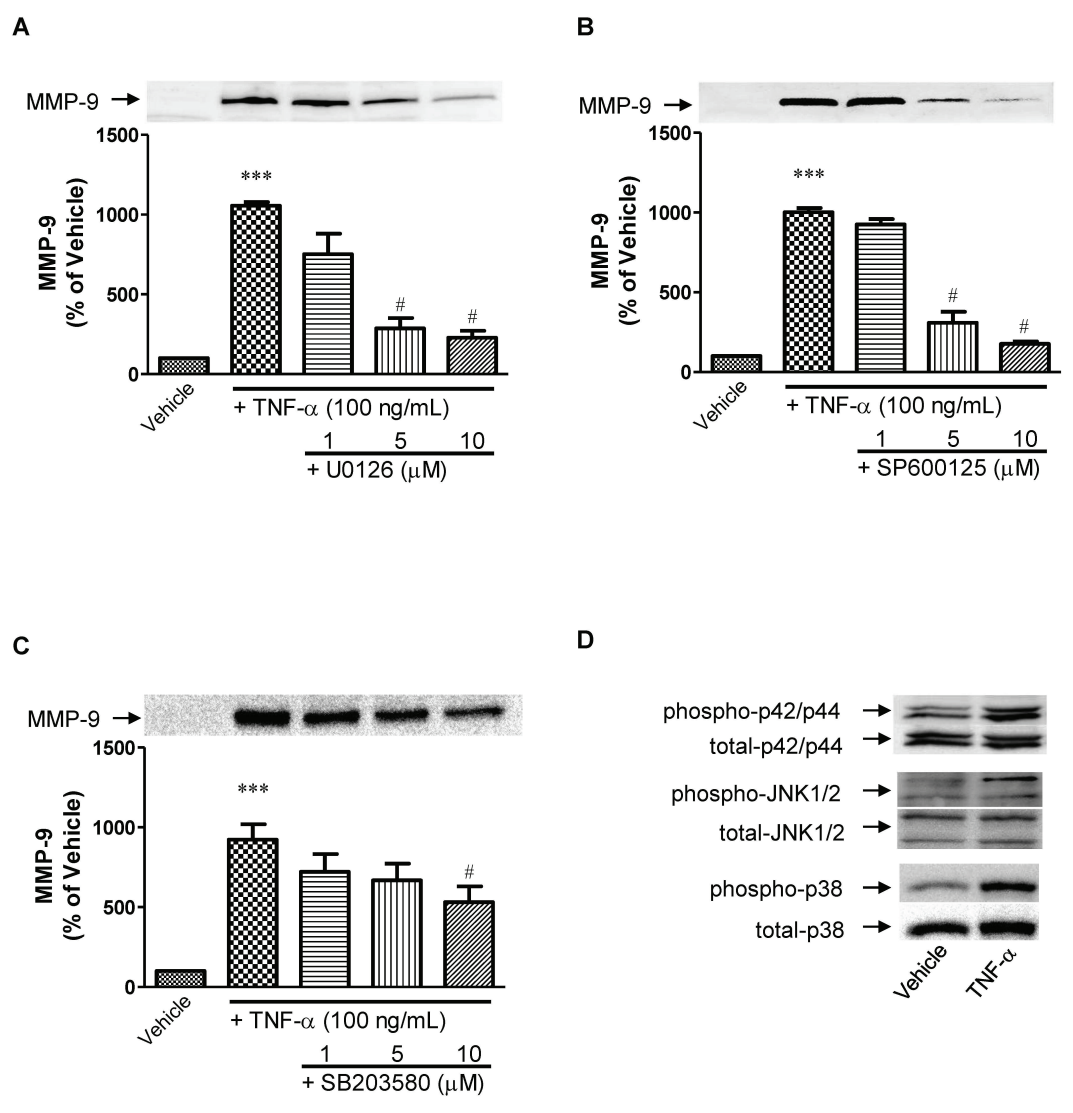
**Involvement of MAPKs (p42/p44 MAPK, JNK and p38 MAPK) in TNF-α-induced MMP-9 release from pericytes**. A, B and C, Cells were pretreated with U0126 (A), SP600125 (B) or SB203580 (C) for 15 min and then incubated with TNF-α (100 ng/mL) for 24 h. Representative western blots (top panels) and densitometric analysis (bottom panels) of MMP-9 levels in the culture media. Band intensities were quantified by scanning densitometry and the data are expressed as percentage of control values (vehicle-treated pericytes). Each bar indicates mean ± S.E.M. for three or more experiments. *** *P *< 0.001, significantly different from vehicle-treated pericytes. ^#^*P *< 0.05, significantly different from TNF-α-treated pericytes. D, Representative images of western blots showing total and phosphorylated p42/p44 MAPK, JNK and p38 MAPK in the cell lysates of pericytes treated with TNF-α(100 ng/mL) for 24 h.

We investigated whether MAPKs are involved in TNF-α-induced MMP-9 release from pericytes. When pericytes were pretreated with a MEK1/2 inhibitor (U0126), a JNK inhibitor (SP600125) and a p38 MAPK inhibitor (SB203580) for 15 min prior to a 24-h exposure to TNF-α (100 ng/mL), TNF-α-induced MMP-9 release was blocked by each inhibitor in a concentration-dependent manner (Figure 3A, B and 3C, respectively). U0126 (10 μM), SP600125 (10 μM) and SB203580 (10 μM) inhibited TNF-α-induced MMP-9 release by approximately 80, 75 and 35%, respectively. TNF-α (100 ng/mL for 24 h) increased the phosphorylation levels of p42/p44 MAPK, JNK and p38 MAPK in pericytes by 102, 75 and 110% of vehicle, respectively (Figure 3D).

### TNF-α induces MMP-9 release from pericytes via the phosphoinositide-3-kinase (PI3K)/Akt cascade

**Figure 4:**
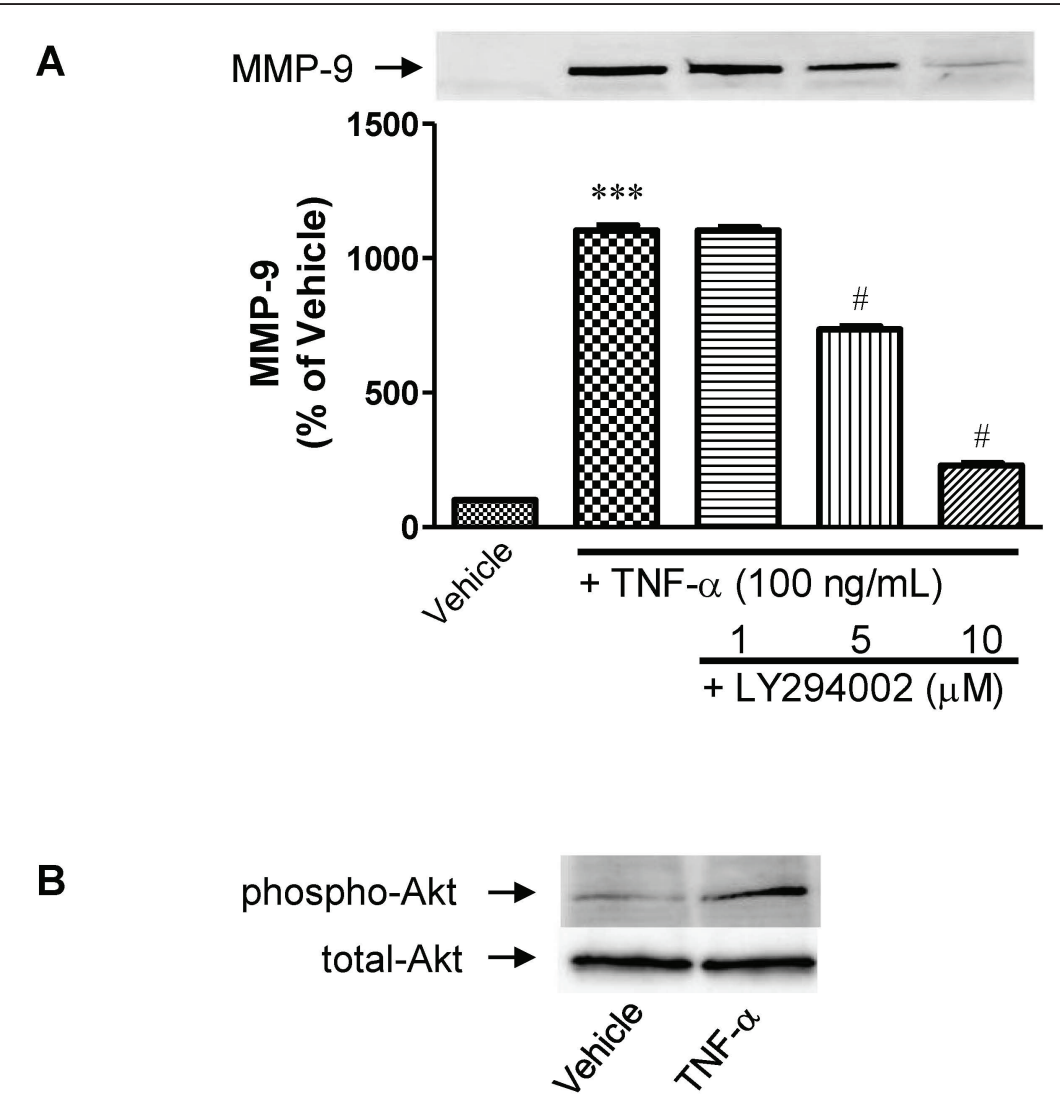
**Involvement of the PI3K/Akt pathway in TNF-α-induced MMP-9 release from pericytes**. A, Effect of PI3K inhibitor (LY294002) on TNF-α-induced MMP-9 release from pericytes. Cells were pretreated with LY294002 for 15 min, and then incubated with TNF-α (100 ng/mL) for 24 h. MMP-9 levels in the culture media of pericytes were assayed by western blot. Band intensities were quantified by scanning densitometry and the data are expressed as percentage of control values (vehicle-treated pericytes). Each bar indicates mean ± S.E.M. for three experiments. *** *P *< 0.001, significantly different from vehicle-treated pericytes. ^#^*P *< 0.05, significantly different from TNF-α-treated pericytes. B, Representative images showing western blots of total and phosphorylated Akt in the cell lysates of pericytes treated with TNF-α (100 ng/mL) for 24 h.

Pretreatment with the PI3K inhibitor, LY294002 (5 and 10 μM), significantly inhibited TNF-α-induced MMP-9 release by approximately 30 and 80%, respectively (Figure 4A). To test whether TNF-α stimulates phosphorylation of Akt, a direct downstream target of PI3K, western blot analysis of pericytes was performed using an anti-phospho-Akt antibody. Phospho-Akt levels were increased in TNF-α-treated pericytes (100 ng/mL for 24 h), compared with vehicle-treated pericytes (Figure 4B).

### Up-regulation of MMP-9 is required for the induction of pericyte migration

**Figure 5:**
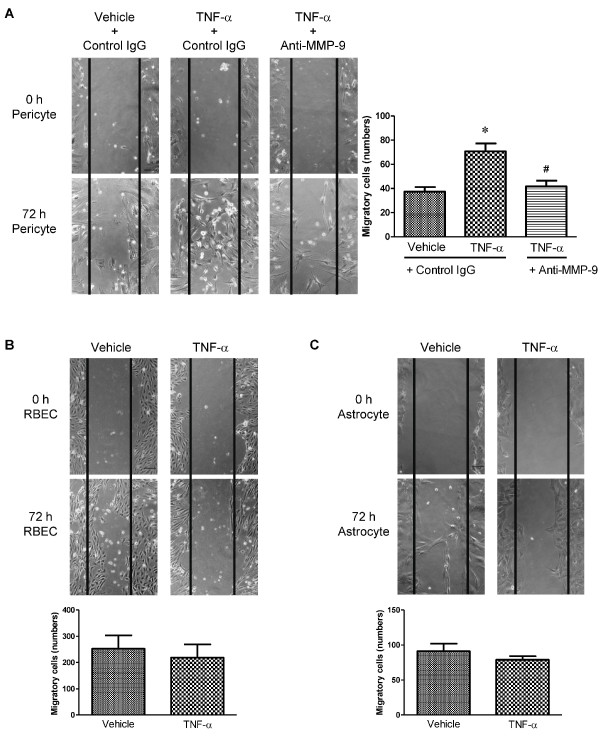
**Effect of TNF-α on migration of pericytes (A), RBECs (B) and astrocytes (C), which constitute the BBB.** Pericytes were treated with TNF-α (50 ng/mL) for 72 h in the presence of mouse IgG or anti-MMP-9 antibody. Astrocytes and RBECs were treated with TNF-α (50 ng/mL) for 72 h. Representative phase-contrast images showing the fixed position in the wound area at 0 and 72 h after scratching (A: left panels; B and C: top panels). The number of cells migrating into the initial wound area was counted at 72 h after scratching and the data are expressed as mean ± S.E.M. for three experiments. (A: right panel; B and C: bottom panels). *P < 0.05, significantly different from vehicle-treated pericytes. ^#^*P *< 0.05, significantly different from TNF-α treated pericytes. Scale Bar = 100 μm.

To evaluate the functional activity of the MMP-9 expression induced by TNF-α, we examined the cellular migration of pericytes using a scratch wound healing assay *in vitro*. Representative images show that TNF-α (50 ng/mL) stimulated pericytes to migrate across the wound edge into the scratched area 72 h after scratching (Figure 5A). The extent of TNF-α-induced pericyte migration significantly increased to 189% of vehicle (Figure 5A). This TNF-α-accelerated migration of pericytes was significantly inhibited and decreased to control levels (vehicle) in the presence of anti-MMP-9 antibody (Figure 5A). TNF-α failed to increase the extent of migration of RBECs and astrocytes (Figure 5B and 5C).

## Discussion

In the present study, our major findings are: (1) at the BBB, brain pericytes are the most sensitive machinery to TNF-α for MMP-9 release; (2) pericytes release higher levels of MMP-9 than BMECs or astrocytes; (3) TNF-α-induced activation of MAPKs (p42/p44 MAPK, p38MAPK and JNK) and PI3K/Akt are vital for increased expression of MMP-9 in pericytes; (4) pericytal MMP-9 promotes cellular migration.

Elevated levels of MMP-9 in the plasma and brain are associated with BBB disruption, leading to an exacerbation of neurodegenerative diseases [25]. MMP-9 is produced in the cells constituting the BBB, including BMECs [26] and astrocytes [27] under pathological conditions. Brain pericytes also produce MMP-9 [28]; however, it has not been clarified whether pericytes release MMP-9 in response to various inflammatory stimuli. In this study, to examine the ability of pericytes to release MMP-9 in response to various inflammatory stimuli, pericytes were treated with TNF-α, IL-1β, IFN-γ, IL-6 and LPS. TNF-α markedly induced MMP-9 release from pericytes. MMP-2 release was not stimulated by TNF-α in these cells, although spontaneous release of MMP-2 was observed. This different response of pericytes to TNF-α between MMP-2 and MMP-9 release suggests that among MMPs, MMP-9 is a potential factor in inducing neuroinflammation in the brain. Interestingly, other inflammatory mediators, including IL-1β, IFN-γ, IL-6 and LPS; did not induce MMP-9 release from pericytes (data not shown). LPS, IL-1β and TNF-α were inducers of MMP-9 in astrocytes and microglia [29,30]. Here, we demonstrate that TNF-α is the cytokine that induces MMP-9 release from pericytes.

Among the three cellular components of the BBB, pericytes produced the highest levels of MMP-9 in response to TNF-α. This TNF-α-induced MMP-9 release increased with time and did not reach a maximum peak for MMP-9 release within 24 h. We evaluated the amount of MMP-9 in the culture supernatants when MMP-9 release was still increasing. Therefore, the possibility that degradation of MMP-9 in culture supernatants had occurred at 24 h after TNF-α-exposure was excluded. These findings suggest that in response to TNF-α pericytes are the major machinery for MMP-9 release from cells constituting the BBB. TNF-α exerts its biological functions by interacting with two members of the TNF receptor superfamily, TNFR1 and TNFR2. We found that TNFR2 expression was 2-fold higher in pericytes compared with astrocytes and RBECs, although TNFR1 expression was not statistically different among these cells. These high levels of TNFR2 expression in pericytes may largely contribute to the TNF-α-induced MMP-9 release from pericytes.

Several studies have indicated that MAPKs (p42/p44 MAPK, p38 MAPK and JNK) and PI3K/Akt pathways are involved in the regulation of MMP-9 expression in endothelial cells [31], vascular smooth muscle cells [32], astrocytes [29] and microglia [33]. TNF-α has been reported to act as an important inflammatory mediator via activation of MAPKs and PI3K/Akt cascades in various cells [34,35]. However, the issue of how the activation of signaling pathways in pericytes results in the induction of MMP-9 is unclear. Here, we demonstrate that stimulation of brain pericytes with TNF-α stimulates phosphorylation of the p42/p44 MAPK, p38 MAPK, JNK and Akt. Inhibition of their activities by their pharmacological inhibitors reduced TNF-α-induced MMP-9 release. These data provide evidence for involvement of the MAPKs and PI3K/Akt pathways in mediating TNF-α-induced up-regulation of MMP-9 release from pericytes. Binding of TNF-α to TNFR1 and TNFR2 activates separate intracellular signaling pathways [35]. We do not present direct evidence to determine whether TNF-α activates MAPKs and PI3K/Akt through TNFR1 and/or TNFR2 in pericytes. Whether the TNF-α receptor subtypes have a role in the mediation of TNF-α-induced MMP-9 release from pericytes is currently under investigation.

MMP-9 plays an essential role in the induction of cellular migration in several cell types [27,36,37]. In the present study, TNF-α enhanced migration of pericytes, but failed to facilitate migration of RBECs and astrocytes. These findings suggest that the amount of MMP-9 induced by TNF-α may be a determinant factor in the acceleration of migration of these cells. Our cell viability assay excluded the possibility that TNF-α stimulates the proliferation of pericytes during the migration test. This TNF-α-induced pericyte migration was suppressed by inhibition of MMP-9 with an inhibitory antibody against MMP-9, indicating that TNF-α stimulates pericytes to enhance migration through MMP-9 release. The proteolytic activity of MMP-9 to degrade extracellular matrices is required for cell migration [38]. The MMP-9 hemopexin domain initiates the intracellular signaling that induces cellular migration; this activity is independent of its proteolytic activity [36,38]. The antibody used in the present study is known to neutralize the hemopexin domain of MMP-9 [39]. These findings raise the possibility that pericytes express receptors for the hemopexin domain of MMP-9 including LDL receptor-related protein 1 (LRP1). In fact, our western blot analysis shows that LRP1 is expressed in pericytes (data not shown). Therefore, TNF-α-accelerated migration of pericytes may be attributed to these activities of MMP-9.

Neuroinflammation has been implicated as a cause of BBB disruption in CNS diseases such as stroke, bacterial meningitis and neurodegenerative diseases [40]. The up-regulation of various inflammatory cytokines under neuroinflammation conditions, especially TNF-α, is known to be a trigger for MMP-9 expression in the brain [41-45]. Previously, we demonstrated that detachment of brain pericytes from the basal lamina is related to disruption of the BBB in LPS-injected mice [11]. Blood-born TNF-α is transported across the BBB [46]. The findings that BMECs secrete TNF-α into the parenchyma [47], and that glial cells express TNF-α in the brain [48,49], are important to understand the mechanism underlying the trigger for pericyte migration. Considering these findings together with our results, it is likely that in neuroinflammatory diseases pericytes at the BBB are very sensitive to TNF-α, resulting in release of MMP-9 through activation of MAPKs and PI3K/Akt signaling pathways. Increased MMP-9 release from pericytes may contribute to two possible pathways that mediate BBB disruption: (1) degradation of extracellular matrices and tight junction proteins of BMECs; (2) enhanced migration of pericytes from microvasculature, appearing as "pericyte loss". Therefore, we propose that pericytes may be able to act as a sensor for neuroinflammatory signals produced by BMECs and brain parenchymal cells (e.g. glial cells), and subsequently release MMP-9 to initiate migration of pericytes. This series of events is an important inflammatory response at the BBB. Further investigations are required to elucidate the pericytes' role during and/or after migration.

## Conclusions

In this study, we demonstrate *in vitro *that pericytes are the major source of MMP-9 release induced by TNF-α at the BBB and that pericyte-derived MMP-9 enhances their migration. Up-regulation of MMP-9 in the cerebral microvasculature probably causes BBB disruption through degradation of tight junctions and extracellular matrices, and subsequent pericyte loss from microvasculature. Therefore, pericytes and pericytal MMP-9 could be attractive therapeutic targets for ameliorating BBB dysfunction in neuroinflammatory diseases.

## List of abbreviations

ANOVA: analysis of variance; BBB: blood-brain barrier; BMECs: brain microvascular endothelial cells; BSA: bovine serum albumin; DMEM: Dulbecco's modified Eagle's medium; DMEM/F12: DMEM/Ham's nutrient mixture F-12 medium; FBS: fetal bovine serum; IFN-γ: interferon-γ; IL-1β: interleukin-1β; IL-6: interleukin-6; JNK: c-Jun N-terminal kinase; LPS: lipopolysaccharide; LRP1: LDL receptor-related protein 1; MAPK: mitogen-activated protein kinase; MMPs: matrix metalloproteinases; PDS: plasma-derived serum; PI3K: phosphoinositide-3-kinase; RBECs: rat brain microvascular endothelial cells; TNF-α: tumor necrosis factor-α; TNFR1: TNF-α receptor 1; TNFR2: TNF-α receptor 2.

## Competing interests

The authors declare that they have no competing interests.

## Authors' contributions

FT designed the study, performed the bulk of the experiments, analyzed all data, and wrote the manuscript. SD designed the study, performed the migration assay, and critically reviewed the manuscript. JM, TM, TW, EH and HM performed the western blot analysis, gelatin zymography, and the preparation of primary cultures. HT participated in the migration assay, data analysis, and the preparation of primary cultures. MK, TN and AY helped with western blot and data analysis. YK critically reviewed the manuscript and supervised the entire research project. All authors have read and approved the final version of this manuscript.
